# Enhanced Fluid Mixing in Microchannels Using Levitated Magnetic Microrobots: A Numerical Study

**DOI:** 10.3390/mi16010052

**Published:** 2024-12-31

**Authors:** Ali Anil Demircali, Abdurrahim Yilmaz, Huseyin Uvet

**Affiliations:** 1 Department of Metabolism, Digestion, and Reproduction, Faculty of Medicine, Imperial College London, London SW7 2AZ, UK; a.demircali@imperial.ac.uk; 2 Mechatronics Engineering Department, Yildiz Technical University, Istanbul 34349, Turkey

**Keywords:** micromixer, numerical analysis, magnetic levitation, microfluidics, mixing optimization

## Abstract

The efficient mixing of fluids at microscale dimensions presents challenges due to the dominant laminar flow regime which restricts convective mixing. This study introduces a numerical analysis of a novel microrobotic mixing system with a levitated propeller robot, driven by magnetic fields, within a Y-shaped microchannel with a square cross-section (500 × 500 μm). Our research investigates the fluid mixing effectiveness facilitated by the microrobot through various levitation heights and orientations to enhance the mixing index (MI). This index is tested under different conditions by leveraging the dynamics of the propeller robot, characterized by adjustable roll and pitch angles and varying levitation heights. The numerical simulations, conducted using COMSOL^®^ (Finite Element Method, FEM) software, integrate Maxwell’s equations for magnetic field interaction with momentum and transport-diffusion equations to analyze fluid dynamics within the microchannel. Results indicate that the propeller robot can achieve an MI of up to 98.94% at a 150 μm levitation height and 1500 rpm propeller speed within 3 s. Additionally, the study examines the impact of propeller speed, Reynolds number, and robot length on mixing performance, providing comprehensive guidance for optimizing microscale fluid mixing in lab-on-a-chip applications.

## 1. Introduction

Recent advancements in Lab-on-a-Chip (LoC), micro-electromechanical systems (MEMS), and micromixer systems highlight the growing interest in these technologies across engineering, biotechnology, and medicine due to their cost-effectiveness, ease of production, and repeatability [1,2,3]. Microfluidic devices condense traditional laboratory functions into compact chips, enabling detection, mixing, synthesis, and separation at micro- and nano-scales. These systems offer significant advantages in chemical and biomedical analysis, including speed, precision, and minimal reagent use [4,5,6]. Within microfluidics, micromixers are fundamental for achieving efficient fluid mixing and are broadly classified into passive and active types, depending on whether external energy sources are required.

Passive mixers operate without external energy and can be manufactured cost-effectively using methods such as lithography [7]. The mixing process in these devices often involves structural modifications within the microchannel to alter fluid flow velocities, such as introducing obstacles [8], implementing sudden constrictions and expansions [9], curving channels [10], or using lamination techniques [11] to promote turbulence. These modifications enable efficient mixing by creating secondary flows and elongating the fluid interface. Optimized split-and-recombine techniques, for example, achieve high mixing efficiencies by leveraging these structural characteristics [12,13]. The channel geometry thus plays an essential role in the passive performance of the micromixer, where a series of repetitive mixing units can increase efficiency by stretching the fluid interface through fluid-geometry interactions. Effective designs often feature serpentine channels, which induce secondary flows to further enhance mixing [14,15,16].

However, the low Reynolds number typical in microfluidic devices limits the feasibility of turbulent mixing, making these systems highly dependent on slower, diffusion-driven processes to achieve adequate mixing, often requiring longer channels [17,18]. To address this limitation, researchers have introduced obstacles within the channel, which extend the fluid-fluid interface, enhancing the mixing efficiency, even in short channel lengths, despite the associated increase in pressure drop [19,20,21,22]. Additionally, combining serpentine channels with embedded obstacles has proven to be a powerful approach, effectively merging the benefits of both strategies to achieve even higher efficiencies [23].

Active mixers, on the other hand, require various energy sources, leading to different production techniques such as pressure [24], acoustics [25,26,27,28], magnetic fields [29,30,31,32,33,34,35,36,37,38,39,40,41], and electric fields [42,43,44]. These mixers provide precise control over mixing rates and ratios at the channel exit, making them ideal for applications that demand rapid response or high-volume mixing, such as drug delivery, diagnostics, and biochemical assays [45]. A key benefit of active mixers is their capability to adjust mixing efficiency (MI) in real time, which can significantly enhance process control. For instance, magnetic actuation is used in microsystems with micro-stirrers that generate circulation loops, effectively reducing mixing time and achieving higher MI at rotor speeds between 100 and 600 rpm [46]. This real-time capability can incorporate permanent magnets [33] or electromagnets [47] to mix ferrofluids or even manipulate magnetic particles for specific biomedical applications [48]. Innovative methods, like creating thermal bubbles or freezing microbeads within the flow, further expand the strategies available for efficient fluid mixing, adding versatility in design for application-specific needs [49].

Magnetic mixing has emerged as a powerful approach in microfluidics, with various strategies demonstrating its potential. Ballard et al. achieved mixing efficiencies between 90% and 95% by utilizing orbiting magnetic microbeads, which generate strong convective flows through magnetic actuation [32]. Liu et al. introduced a magnetically actuated cilium-based micromixer that achieved approximately 80% mixing efficiency, demonstrating rapid performance enhancement [35]. Saroj et al. utilized magnetic beads in sessile droplet setups, optimizing mixing through controlled actuation currents and frequencies, which significantly improved fluid homogeneity [36]. Nouri et al. enhanced ferrofluid mixing efficiency up to 90% by increasing nanoparticle concentrations and optimizing field strengths in microchannels [33]. Hejazian et al. demonstrated mixing efficiencies of up to 88% by employing magnetoconvection mechanisms in short microchannels [34]. Boroun et al. improved micromixing using low-frequency rotating nanoparticles, achieving significant efficiency gains in liquid–liquid mixing applications [37]. Usefian et al. achieved 83% efficiency in 3D microfluidic mixing using non-uniform magnetic fields, with inverse correlations observed between efficiency and inlet velocity [30]. Shanko et al. achieved enhanced mixing in sinusoidal microchannels by optimizing magnet distance, resulting in an efficiency improvement of up to 73% [38]. Bahrami et al. further improved mixing efficiency by 24% through increased nanoparticle volume fractions and precise control over non-uniform magnetic fields [39]. Buglie et al. achieved up to 99.42% efficiency using magneto-hydrodynamic mechanisms in cylindrical micromixers, showcasing one of the highest efficiencies in the field [50]. Broeren et al. demonstrated reversible and on-demand mixing through actuated magnetic microwalls, leveraging the inertial effects in meandering flow paths to enhance efficiency [51]. Naghash et al. reported efficiencies of up to 90% in programmable magnetic systems by the varying movement patterns of microballs [52]. Saadat et al. highlighted the advantages of external varying magnetic fields, achieving up to 97.4% efficiency in lab-on-chip systems [40].

While these studies showcase the advancements in magnetic mixing, the accuracy of mixing efficiency predictions often depends on the methods used for simulations and experiments. In both active and passive mixer systems, mixing calculations are typically performed under room conditions using the finite element method (FEM) for steady-state scenarios. These simulations often assume non-Newtonian and incompressible fluid properties [53,54,55], which can lead to overestimations in simulated efficiency compared to experimental results. Optimizing channel size, geometry, and flow rate improves MI, often using two-dimensional (2D) FEM for reduced computation time [56]. However, 2D and 2D axisymmetric models cannot capture flow variations across channel heights, leading to potential inaccuracies in performance predictions. Three-dimensional (3D) analyses address these issues, accounting for fluid velocity variations and local Re values to provide a more accurate representation of mixing dynamics [57].

A 3D analysis of a microrobot-based active micromixer introduced a system with a rotor embedded in a microfluidic chip, enabling mixing at various rotor heights but excluding magnetic field influence, which complicates modeling due to nonlinear interactions [13]. Practical application requires leak-proof chip designs, and our work proposes a simpler microrobotic configuration that eliminates leak-proofing and integrates seamlessly into microfluidic chips. Another study demonstrated a mobile microrobot system driven by a Helmholtz coil for mixing, though it faced scalability and control complexity issues [43]. Our configuration, featuring two microrobots with one as a motor-driven actuator, allows adjustable levitation and orientation, representing a significant advance in microfluidic mixing technology.

In this study, we introduce a novel untethered microfluidic mixer system utilizing two identical microrobots that interact magnetically, with one acting as a propeller robot whose height is precisely adjusted through magnetic levitation. This levitation mechanism significantly reduces surface friction, enabling higher operational speeds and improved control over fluid dynamics, which are challenging in conventional tethered or fixed-position mixers. By simulating various roll and pitch orientations, we replicated the movement of a curved propeller blade without requiring complex 3D production techniques, streamlining fabrication, and enhancing scalability. Critical parameters such as maximum drag force, torque, and rotational speeds were calculated across different configurations to determine their effects on mixing efficiency (MI). Performance testing at three distinct sections of the channel outlet, using data from 13,556 points over a 3 s interval, showed exceptional mixing efficiency, achieving up to 99.16% MI at 2000 µm from the chamber. These results highlight our microrobot levitation system’s capability for efficient mixing in compact, short-channel designs suitable for diverse applications.

## 2. Materials and Methods

### 2.1. Microchannel Design

**Figure 1:**
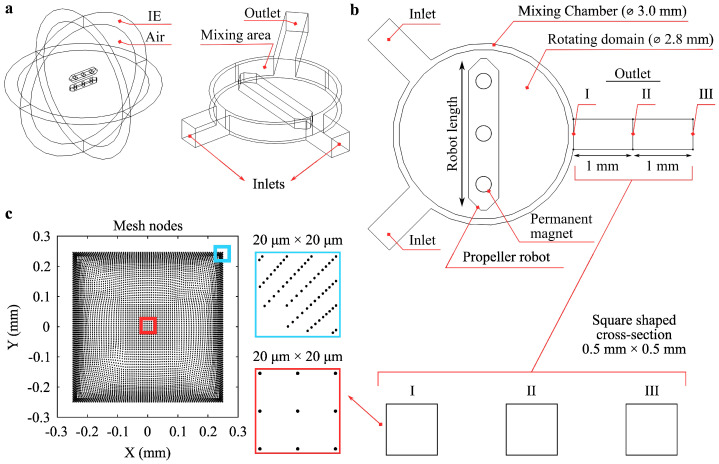
Schematic of the active mixing system with design parameters. (**a**) Overview of the system setup, where two identical microrobots are positioned within a spherical air domain enclosed by an Infinite Element (IE) boundary. The Y-shaped channel has a square cross-section, featuring two inlets and one outlet, and a single propeller robot at the center that rotates to mix incoming fluids. (**b**) Top view of the Y-shaped channel, highlighting the mixing chamber (3.0 mm diameter), rotating domain (2.8 mm diameter), and outlet sections I, II, and III, each spaced 1 mm apart. (**c**) Mesh node distribution in a 0.5 mm × 0.5 mm cross-section of the channel, with detailed views of center (red) and corner (blue) nodes in 20 µm × 20 µm regions.

The active mixing system was designed around a Y-shaped microfluidic channel with a 0.5 mm × 0.5 mm square cross-section, featuring two fluid inlets and one outlet (Figure 1). Fluid was introduced through the inlets, with concentrations set at 0 mol/m^3^ and 1 mol/m^3^ to create a gradient aimed at achieving a uniform 0.5 mol/m^3^ concentration at the outlet, indicative of 100% mixing index (MI). A central mixing area, enclosed by an Infinite Element (IE) boundary to simulate an unbounded domain, provides a spherical air environment where two identical microrobots are positioned (Figure 1a). These microrobots contain N52-grade permanent magnets that generate a magnetic field, enabling levitation and rotation within the channel. The magnetic forces between the microrobots control their orientation and allow the propeller robot to rotate, inducing mixing as it interacts with incoming fluids from the inlets.

The microchannel includes a primary mixing chamber (3.0 mm in diameter) and an effective rotating domain (2.8 mm in diameter), where the microrobots are actively engaged in fluid agitation (Figure 1b). To monitor mixing performance, three sections (labeled I, II, and III) were defined along the outlet channel, each spaced 1 mm apart, to evaluate the mixing at specific points immediately after the mixing area, at the midpoint, and at the outlet end, assessing the effect of diffusion on MI.

To achieve an accurate representation of flow dynamics, the channel geometry was discretized using a fine mesh, with a total of 13,556 sample points in the COMSOL simulation (Figure 1c). Mesh density was higher in critical regions, including near the center and corners, to capture detailed fluid flow and concentration gradients, especially close to channel walls where velocity gradients are most significant. Sample points were distributed within 20 µm × 20 µm regions, represented by the blue- and red-marked rectangles in Figure 1c, to ensure high-resolution mixing calculations. The boundary element mesh along the channel walls factored in gravity and surface tension effects.

To evaluate the efficiency of mixing, the microrobot system was analyzed in the subsystems, including magnetic field, fluid flow, and transport of diluted species. Each subsystem was modeled and simulated in 3D using COMSOL Multiphysics software v6.0 [58]. The Magnetic Fields No Currents (MFNC) module was used for magnetic torque analysis, while the Moving Mesh (Rotating Domain) and Laminar Flow (SPF) modules handled drag force calculations and fluid dynamics. Angular displacement and torque relationships were examined through Moving Mesh (Rotating Domain), Laminar Flow, and Global Equations (GE) modules. Post-processing yielded maximum torque and rotational speeds at various levitation heights. Table 1 lists the boundary conditions used in COMSOL for solving fluid flow problems.

### 2.2. Magnetic Field

Magnetic field analyses calculated the attractive force among three permanent magnets within the microrobots. The magnetic field was modeled using the following Maxwell’s equations:

(1) ∇×H=0

where H represents the magnetic field distribution (A/m). The relationship between the magnetic flux density B (W/m^2^) and H is given by the following:

(2) $$B=\mu_{0}\left(M+H\right)$$

where $\mu_{0}$ is the vacuum permeability and M the magnetization (A/m). The magnetic scalar potential $V_{m}$ (A) was determined using the magnetic fields, no currents interface, leading to the following:

(3) $$-\nabla (\mu_{0}\nabla V_{m}-\mu_{0}M)=0$$

This equation assumes a constant magnetic scalar potential. The attractive magnetic force $F_{m}$ exerted by the actuator robot on the propeller robot is then expressed as follows [59]:

(4) $$F_{m}=\frac{\mu_{r}-1}{2\mu_{0}\mu_{r}}{\;\int \int \int \;}_{V}\nabla B^{2}\;dv$$

where $\mu_{r}$ is the relative permeability. The torque produced by the magnet in an external field is determined by the following:

(5) τ=αM×B

(6) $$T_{e}=\ell \int \sum_{i=1}^{N}\left(dF_{yi}r_{xi}-dF_{xi}r_{yi}\right)$$

where $T_{e}$ is the total torque, α is the anisotropy, *ℓ* is the depth of the domain (in the z-direction), and *r* is the position vector linking the rotation axis to the element dT. Here, dF is the elemental force calculated over *N*, the number of elements with surface dT. $dF_{yi}$ and $dF_{xi}$ are the elemental forces acting along the y and x directions, respectively, while $r_{xi}$ and $r_{yi}$ are the components of the position vector *r*, defined by the rotation axis and the midpoint of dT.

### 2.3. Fluid Flow

The steady-state incompressible Navier–Stokes equations were used, with simulations solved alongside the continuity equation [60]. The Partial Differential Equation (PDE) model was used to employ the stationary incompressible Navier–Stokes equations as follows:

(7) $$\rho \left(\frac{\partial u}{\partial t}+\left(u\cdot \nabla \right)u\right)=-\nabla p+\mu \nabla^{2}u+F$$

Here, ρ represents the density of the fluid (kg/m^3^), u the velocity field vector (m/s), *p* the pressure (Pa), μ the dynamic viscosity (Pa·s), and F the external body forces (N/m^3^). Mass conservation for fluid flow is based on the following continuity equation:

(8) ∇·u=0

The modeling of mixing processes for both Newtonian and non-Newtonian fluids is governed by the continuity, momentum, and energy equations. The energy equation, which represents the conservation of energy, is presented as follows:

(9) $$\frac{\partial \rho e}{\partial t}=\nabla \cdot \left(\rho eu-\tau u+q\right)$$

Here, *e* denotes the energy per unit mass, τ the stress tensor, and q the heat flux vector. The Reynolds number, Re, which characterizes the flow regime (laminar or turbulent), is defined as follows:

(10) $$Re=\frac{\rho U_{\mathrm{avg}}D_{h}}{\mu }$$

where $U_{\mathrm{avg}}$ is the average velocity (m/s), $D_{h}$ the hydraulic diameter (m), and μ the dynamic viscosity (Pa·s).

### 2.4. Transport of Diluted Species

The Laminar Flow (SPF) and Transport of Diluted Species (TDS) physics interfaces were utilized. The TDS interface handled the mixing of two dilute species, A and B, within the main circular area of the channel. These species entered the device through separate inlet ports at equal concentrations, diffusivities, and flow rates. The transient behavior of the mixing system was observed through time-dependent studies over 3 s at room conditions. The fluid was incompressible and Newtonian, with a density ($\rho_{w}$) of 1000 kg/m^3^ and a dynamic viscosity ($\mu_{w}$) of 0.001 (Pa·s). The concentration distribution of the species is determined by the following convection-diffusion transport equation:

(11) $$\frac{\partial c_{i}}{\partial t}+\nabla \cdot N_{i}+u\cdot \nabla c_{i}=R_{i}$$

(12) $$N_{i}=-D_{i}\nabla c_{i}$$

Here, $N_{i}$ represents the molar fluxes, u the characteristic flow speed, $R_{i}=r_{i}/M_{i}$ the rate of moles of species *i* produced per unit volume per unit time, $c_{i}$ the species concentration, and $D_{i}$ the diffusion coefficient. The MI of the micromixers was evaluated using the Mixing Index (MI) [61,62,63,64], calculated as follows:

(13) $$\mathrm{MI}\left(\%\right)=\left(1-\frac{|{\bar{c}}_{i}-c_{\mathrm{ref}}|}{c_{\mathrm{ref}}}\right)\times 100$$

where ${\bar{c}}_{i}$ is the mean concentration at each sampling node *i*, and $c_{\mathrm{ref}}$ is the reference concentration when the fluids are completely mixed, assumed to be 0.5. An MI of 1 corresponds to perfect homogeneity in this system. The standard deviation (SD) as a percentage by referencing the target mixing value as follows:

(14) $$\sigma \left(\%\right)=\left(1-\frac{|\sigma \left(c_{i}\right)-c_{\mathrm{ref}}|}{c_{\mathrm{ref}}}\right)\times 100$$

The homogeneity of the mixing was evaluated using the Homogeneity Index (HI), calculated as follows:

(15) $$\mathrm{HI}\left(\%\right)=\left(1-\frac{\sigma (c_{i})}{{\bar{c}}_{i}}\right)\times 100$$

where ${\bar{c}}_{i}$ is the mean concentration of the mixed species at each sampling node *i*, and $\sigma (c_{i})$ is the standard deviation of the concentration at the same nodes. The HI ranges from 0% to 100%, where 100% indicates perfect homogeneity (complete mixing) and values closer to 0% indicate less homogeneity.

## 3. Numerical Analysis

In COMSOL, boundary conditions and key parameter settings were selected to reflect realistic microfluidic conditions and accurately assess mixing efficiency (MI) (Table 1). Inlet concentrations, set at $c_{1}=0$ and $c_{2}=1$ mol/m^3^, provided a high gradient to challenge the mixing capability of the system. A no-slip boundary ($u_{\mathrm{wall}}=0$ mm/s) was applied along the channel walls to simulate realistic frictional effects, important in microfluidic flows. A fixed pressure of P = 0 Pa was applied at the outlet to represent flow exiting to atmospheric conditions, ensuring a consistent reference for fluid flow. We used a species flow rate ratio of $R_{AB}=1$ for symmetric fluid entry, allowing internal dynamics to drive MI. Remanent flux density (B=1.4 T) ensured the stable magnetic control of the microrobot while preventing excess force that could disrupt its orientation. To cover all low, intermediate, and high Reynolds number regimes [65] comprehensively, we selected a range from 0.01 to 1000, which aligns with similar works’ Re ranges [66,67,68,69]. Additionally, propeller speeds from 0 to 6000 rpm were employed to evaluate the rotational effects on the Mixing Index (MI), ensuring the results reflect diverse flow dynamics within the specified operational range. Robot lengths from 0.75 to 2.5 mm were tested to evaluate size-dependent drag impacts on mixing efficiency, essential in optimizing microrobot dimensions within microfluidic channels.

### 3.1. Magnetic Field and Propeller Velocity

**Figure 2:**
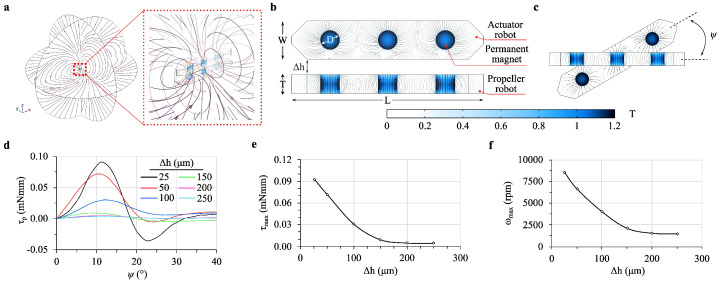
Schematic of the active mixing system with design parameters. (**a**) The magnetic field distribution generated around two identical microrobots within the mixing system. The inset shows a close-up view of the concentrated field lines between the actuator and propeller robots, illustrating the magnetic interactions that facilitate mixing. (**b**) Close frontal view of the robots with magnetic field force lines. (**c**) The angular difference, ψ, between the actuator and propeller robots during rotation, varies with fluid and actuator speeds. (**d**) Torque, $\tau_{p}$, on the propeller robot as a function of ψ. (**e**) Relationship between maximum torque $\tau_{max}$ and levitation height, Δh, showing an exponential decrease in torque with increasing Δh. (**f**) Maximum propeller speeds, $\omega_{max}$, indicating a range of 1501.4–8602.4 rpm.

The levitation height of the propeller robot is adjustable based on the vertical distance between the robots. Different drag forces and magnetic forces exerted on the propeller robot affect the maximum achievable rotational speeds. To compare the effect of different levitation heights on mixing rates under consistent conditions, it is necessary to keep the robots’ speeds identical. Drag force and maximum torque values were calculated for each levitation height to determine the maximum rotational speed of the propeller robot. The analysis was performed in two stages using different setups for magnetic analyses (Figure 2a) and for examining the drag force and MI within a Y-shaped microfluidic channel (Figure 2b), where a microrobot centrally located mixes fluids from two inlets. The model in Figure 2a, featuring two identical microrobots placed at the center of an air domain with a specific levitation height Δh, was used to calculate the magnetic torque produced in the propeller robot. The surrounding Infinite Element (IE) domain enhanced the precision of these calculations. The next phase involved examining the drag force on the microrobot surface. The perpendicular placement of robots optimizes lateral force benefits, contrasting with parallel arrangements (Figure 2b).

As the propeller robot rotates in the fluidic chamber, it experiences significantly more drag force compared to the actuator robot, which is driven by a motorized system in the air; an angular offset (ψ) was expected between these robots [3,59]. The generated torque on the propeller robot was calculated using rotating robots to each other (Figure 2c), with results shown in Figure 2d. The torque values are inversely proportional to the levitation height Δh, influenced by lateral forces on the propeller’s magnets. The relationship between maximum torque values and Δh shows a more linear trend for Δh=150 μm and higher, as depicted in Figure 2e. The maximum speed values show characteristics similar to those of torque values, as shown in Figure 2f. Maximum speed calculations, which employed a stop condition according to Global Equation (GE) physics to balance magnetic torque ($\tau_{M}$) against drag torque ($\tau_{D}$), determine the propeller’s maximum rotational speed at the given levitation height. To maintain equal conditions for MI across different levitation heights, subsequent analyses were conducted at a constant propeller speed of 1500 rpm.

**Figure 3:**
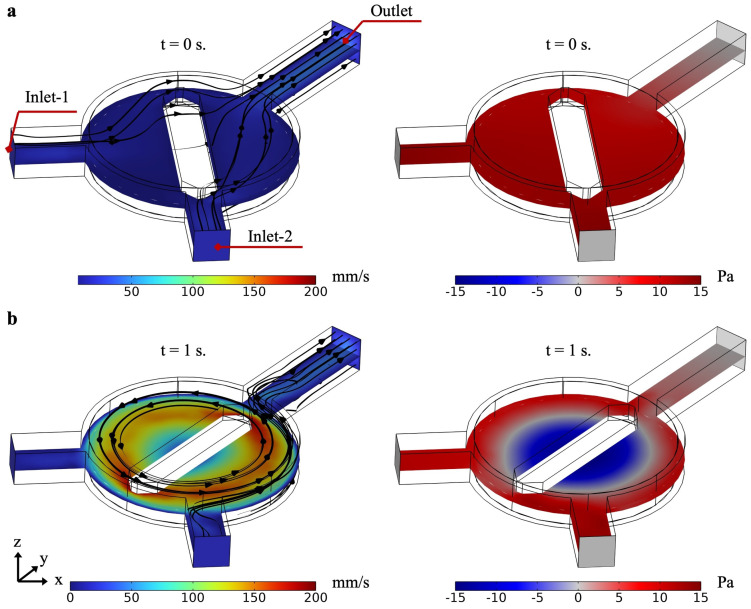
Flow velocity and pressure distribution in the mixing chamber at different times. (**a**) Initial state (t = 0 s) showing velocity magnitude and pressure distribution as fluid enters through Inlet-1 and Inlet-2. The velocity plot (left) indicates initial flow patterns with high speeds near the inlets, while the pressure plot (right) displays a symmetric pressure distribution. (**b**) After 1 s (t = 1 s), flow velocity (left) and pressure distribution (right) reflect the development of rotational flow within the chamber, driven by the propeller robot. Velocity gradients in the mixing area indicate effective fluid mixing, and pressure differentials illustrate the dynamic impact of rotation.

Additionally, the fluid flow and pressure distribution in the mixing chamber were analyzed to observe the dynamic impact of the propeller’s rotation on mixing. The initial state (t = 0 s) is shown in Figure 3a, where velocity magnitude and pressure distribution indicate symmetrical patterns as fluids enter through Inlet-1 and Inlet-2. After 1 s (t = 1 s), flow velocity and pressure distribution reflect the development of rotational flow within the chamber, driven by the propeller robot (Figure 3b). Velocity gradients in the mixing area suggest effective fluid mixing, while pressure differentials illustrate the influence of the rotating propeller on the overall flow dynamics.

### 3.2. Levitation Height and Propeller Orientation

**Figure 4:**
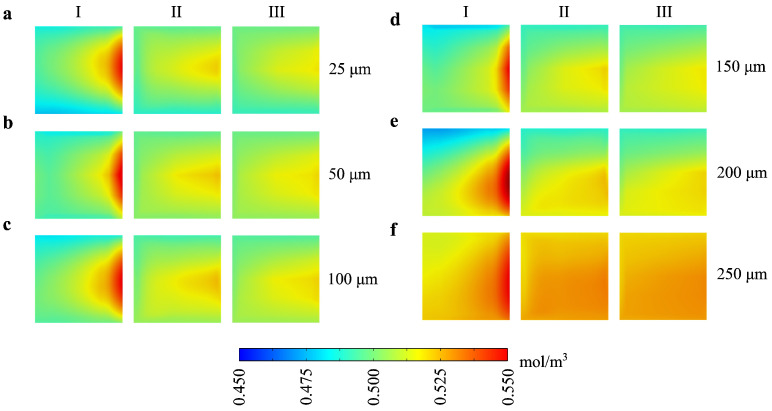
Mixing Index (MI) distributions at different levitation heights of the propeller robot, showing concentration profiles across three sections (I, II, III) at the outlet. Each row (**a**–**f**) corresponds to a specific levitation height (25, 50, 100, 150, 200, and 250 µm, respectively) and demonstrates the concentration distribution from the inlet toward the outlet. The color bar (0.45–0.55 mol/m^3^) represents the concentration levels, where red indicates higher mixing efficiency and blue represents lower mixing efficiency. As levitation height increases, the mixing efficiency generally decreases, as indicated by lower concentration gradients across sections I, II, and III.

Figure 4 illustrates how levitation height affects the Mixing Index (MI) across sections I, II, and III. At lower levitation heights (e.g., 25 µm and 50 µm), the concentration profiles show a sharper gradient across the sections, indicating effective mixing due to the propeller robot’s proximity to the fluid. This close positioning increases fluid turbulence and interfacial area, thereby enhancing mixing efficiency. As the levitation height increases (e.g., 150 µm to 250 µm), the concentration gradients become more uniform, with MI values decreasing across the sections. This reduction in mixing efficiency at higher levitation heights suggests that the propeller’s influence on the fluid diminishes as it moves further from the mixing area, leading to lower turbulence and weaker fluid interactions.

Building on the observed effects of levitation height on MI, we next examined the impact of the angled orientations of the propeller robot. To assess the effects of roll and pitch (phi) orientation on MI, the actuator robot was rotated around the yaw axis and tilted along the roll axis at an optimal levitation height of 150 μm, chosen to minimize the risk of the robot contacting the channel surfaces. Simulations were conducted in $0.5^{\circ }$ increments from $0^{\circ }$ to $4^{\circ }$, totaling nine simulations over a 3 s duration. Initial conditions were calculated as in Section 3.1 using a Frozen Rotor study. The same simulations for the roll axis orientation were replicated for the pitch axis to fully explore the effects of both orientations.

**Figure 5:**
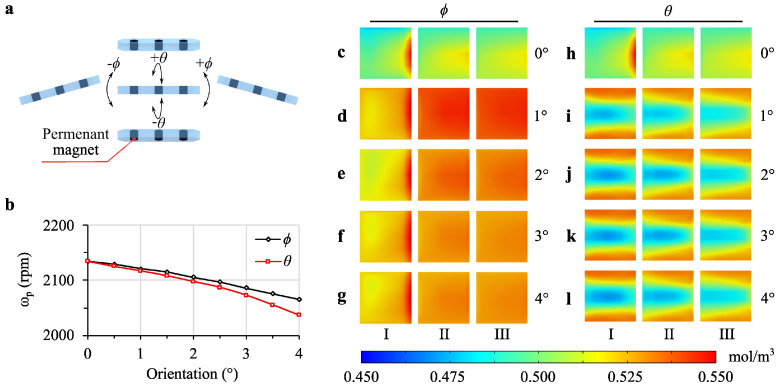
The effect of different orientation and angle values of the propeller robot at a levitation height of 150 μm on mixing is illustrated. (**a**) The orientation capabilities of a propeller robot positioned parallel to the surface, expressed as the roll angle, ϕ, and pitch angle, θ, controlled by the actuator robot. (**b**) Speed values during the robot’s orientation at different angles, with the speed values corresponding to $0^{\circ }$–$4^{\circ }$ for ϕ (in black) and θ (in red). Visuals of the concentrations at 3 s for the ϕ angle are shown in (**c**–**g**), and for the θ angle in (**h**–**l**), respectively, for angles $0^{\circ }$ to $4^{\circ }$ with a $1^{\circ }$ interval. A common color bar with limits of 0.450–0.550 mol/m^3^ is used for both groups.

Figure 5 shows a propeller robot parallel to the surface at its center, with the three permanent magnets inside depicted in a semi-transparent robot body. The robot’s orientation changes along its short edge (longitudinal) and its long edge (lateral) were represented by the roll angle (ϕ) and the pitch angle (θ), respectively. The speed changes, as shown in Figure 5b, were calculated in the propeller robot resulting from these orientations, with black and diamond markers for ϕ and red square markers for θ. Additional pitch movement alters the surface area actively interacting with the fluid, resulting in a greater reduction in speed. Visual representations of the concentrations at 3 s for orientations ϕ (Figure 5c–g) and θ (Figure 5h–l) are shown for angles $0^{\circ }$–$4^{\circ }$, using a common color bar range of 0.450–0.550 mol/m^3^.

## 4. Results and Discussion

The propeller robot was levitated and rotated through interactions between its permanent magnets and those in the actuator robot. The maximum torque exerted on the propeller robot increases as levitation height decreases at the same angle difference (Figure 2d). Beyond 250 µm, no significant torque was generated and the highest torque value was observed at ψ= 12.7°, with a value of 0.09 mNmm at 100 µm. We observed a linear relationship between levitation height and both maximum torque and rotational speed up to 150 µm (Figure 2e,f). Beyond 150 µm, significant torque and rotational speeds were not observed. At levitation heights of 25 µm and 100 µm, the torque and rotational speedz were 0.1 mNmm and 8602.4 rpm, and 0.09 mNmm and 7500 rpm, respectively.

### 4.1. MI and HI Importance

The Mixing Index (MI) and Homogeneity Index (HI) are key metrics for evaluating mixing performance in microfluidic systems. A high MI indicates effective overall mixing, while a high HI reflects uniformity in the distribution of mixed components. For example, a high Reynolds number (Re) can result in a high MI but a low HI because rapid flows might not provide sufficient time for uniform mixing, leading to uneven distribution. Conversely, specific orientation angles might yield a high HI but lower MI, showing uniform yet incomplete mixing. Achieving the best mixing performance requires both MI and HI to be high, ensuring thorough and uniform mixing throughout the system. To facilitate comparison of performance, we introduced a new parameter, the quality index, which is a composite measure of MI and HI (i.e., the mean of the sum of the mixing and homogeneity indexes). The quality index further supports these findings, achieving an overall quality index of 97.11% within 500 ms and 98.94% within 3 s at a rotational speed of 1500 rpm. At the outlet of the mixing chamber, the quality index reached 99.11% at 1 cm away and 99.16% at 2 cm away from the chamber after 3 s.

### 4.2. Levitation Height Effect

**Figure 6:**
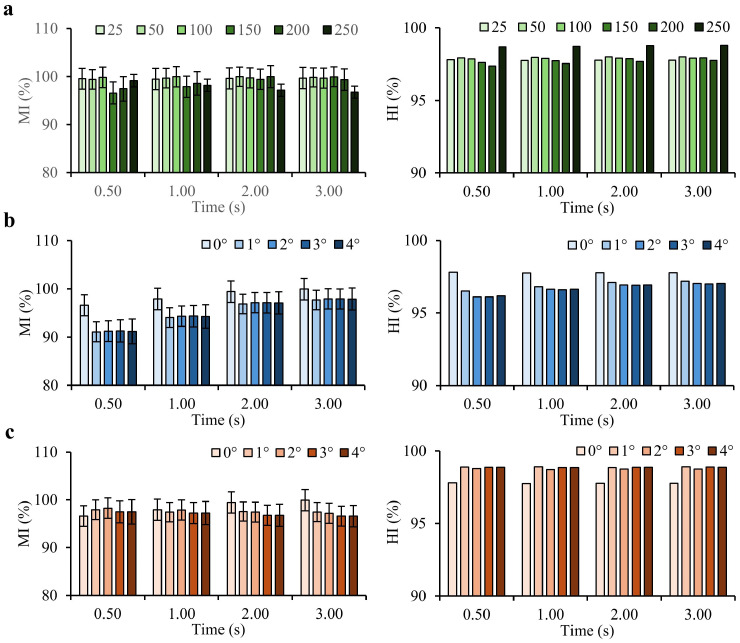
Mixing Index (MI) and Homogeneity Index (HI) as functions of time for different conditions. (**a**) Different levitation heights from 25 to 250 µm at time intervals of 0.5 s, 1 s, 2 s, and 3 s. Initial conditions (0 s) have non-uniform mixing, with data for finer intervals (0, 0.25, 0.5, 1, 1.5, 2, 2.5, 3 s) provided in the Supporting Materials. (**b**) Propeller orientations with pitch angle (θ) from $0^{\circ }$ to $4^{\circ }$ with $1^{\circ }$ intervals. (**c**) Propeller orientations with roll angle (ϕ) from $0^{\circ }$ to $4^{\circ }$ with $1^{\circ }$ intervals. The $0^{\circ }$ orientation shows the best results for both θ and ϕ parameters.

Figure 6a illustrates the relationship between levitation height and both the Mixing Index (MI) and Homogeneity Index (HI) over time (Supplementary Table S1). A levitation height of 100 µm achieves the highest average MI, indicating an optimal balance between torque generation and mixing efficiency. At lower heights (25 and 50 µm), higher initial mixing efficiencies are observed, likely due to the closer proximity of the propeller robot to the chamber floor, which enhances turbulence and fluid interaction. However, these lower heights may risk increased physical interaction with the chamber floor, potentially impacting the long-term stability of the mixing process.

As levitation height increases beyond 100 µm (up to 250 µm), MI and HI values decline. This reduction can be attributed to the increased distance between the propeller robot and the lower fluid layers, reducing the robot’s influence on fluid motion and shear force generation. The diminished fluid interaction at these greater heights results in decreased mixing efficiency, as indicated by the lower MI and HI values. This shows the important role of levitation height optimization in achieving effective mixing while maintaining operational stability. These results identify 100 µm as an optimal levitation height, where the propeller is positioned close enough to induce sufficient turbulence and fluid movement without risking collision or excessive drag forces.

### 4.3. Theta (θ) and Phi (ϕ) Effect

Figure 6b shows the effect of propeller orientations with pitch angle (θ) from 0° to 4° at 1° intervals (Supplementary Table S2). The 0° orientation shows the best results for θ. Rotational speed decreases with increasing pitch angle, with the most significant speed reduction observed for larger angles. This reduction in speed impacts MI negatively, as shown by the lower MI and HI values for higher θ angles. These results suggest that minimizing the pitch angle is beneficial to achieve a higher MI. The larger pitch angles cause greater drag forces, reducing the rotational speed and consequently the MI.

Figure 6c illustrates the effect of propeller orientations with roll angle (ϕ) from 0° to 4° with 1° intervals (Supplementary Table S3). Similar to the pitch angle, the 0° orientation provides the best MI. However, the impact of increasing the roll angle is less pronounced than that of the pitch angle. This suggests that while both angles affect MI, adjustments in pitch angle have a more significant impact than roll angle changes.

### 4.4. Propeller Speed and Inlet Flow Rate

**Figure 7:**
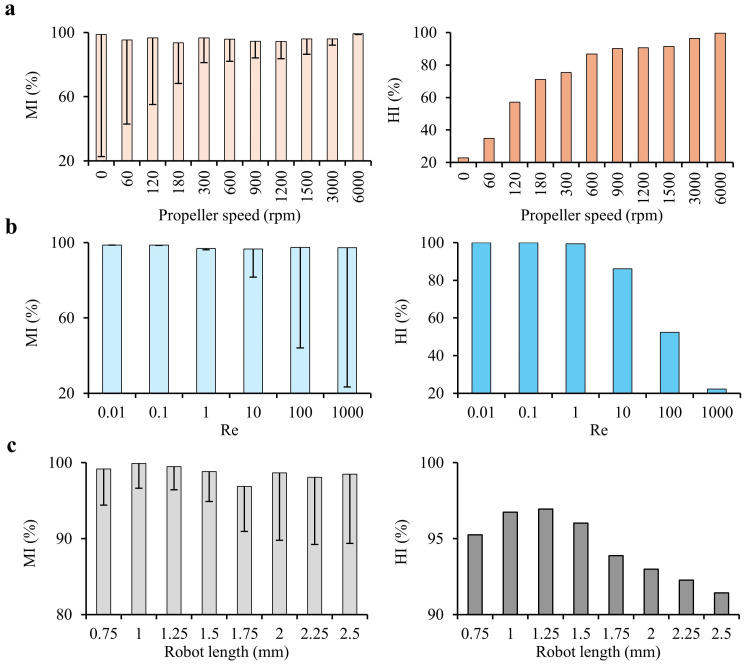
Mixing Index (MI) and Homogeneity Index (HI) for various conditions. (**a**) MI and HI as functions of propeller speed, ranging from 0 to 6000 rpm. A faster rpm reduces the standard deviation (SD%) in MI% and increases HI%, indicating improved MI with higher rotational speeds. (**b**) MI and HI as functions of Reynolds number (Re), ranging from 0.01 to 1000. Higher Re increases SD% as the liquids move too quickly through the channel length, not allowing enough time for effective mixing, resulting in reduced HI%. (**c**) MI and HI as functions of robot length, ranging from 0.75 mm to 2.5 mm. A length of 1.25 mm gives the best result, showing the highest MI, but it has limited space for three magnets in the chamber with a diameter of 3 mm.

As shown in Figure 7a, increasing the propeller speed from 0 to 6000 rpm enhances MI and HI while simultaneously reducing the standard deviation in MI, indicating more consistent and efficient mixing occurs. At the maximum tested speed of 6000 rpm, the highest MI and HI values were achieved, reaching 99.219% and 99.509%, respectively. This trend underscores the direct correlation between rotational speed and mixing efficiency. Higher speeds improve fluid turbulence and interfacial area interactions, which facilitate better homogenization. In contrast, lower speeds are less effective in maintaining consistent mixing across the chamber, as indicated by the higher standard deviation. This analysis highlights the importance of optimizing rotational speed to maximize MI and HI, as faster rpm promotes more uniform concentration distribution throughout the system.

Figure 7b explores the impact of Re, ranging from 0.01 to 1000, on MI and HI. At lower Re values, such as 0.01, the system achieves an MI of 98.767% and an HI of 100%, reflecting nearly ideal mixing conditions. This high efficiency at low Re is likely due to the slower fluid velocity, which allows for sufficient residence time within the mixing chamber for diffusion and advection processes to thoroughly mix the fluids. However, as Re increases, there is a marked decline in both MI and HI. For instance, at Re = 1000, MI falls to 97.264% and HI drastically decreases to 22.276%, indicating incomplete mixing. This decline at higher Re can be attributed to the rapid flow rate, which reduces the time available for fluids to interact and homogenize, causing them to pass through the chamber with insufficient mixing. Consequently, balancing the flow rate (and hence the Re) is essential in achieving effective mixing, especially in applications where precise control over concentration gradients is critical.

Together, the results indicate that both high rotational speed and controlled Re are essential for maximizing mixing efficiency in microfluidic systems, while increasing propeller speed enhances MI and HI by promoting turbulence and consistent fluid interaction, keeping Re at lower levels (e.g., Re = 0.01) and ensuring that fluids remain within the chamber long enough for complete mixing. The optimal configuration, therefore, involves a high propeller speed (close to 6000 rpm) combined with a low Re, which leverages both enhanced turbulence and adequate residence time. Deviations from this balance, such as excessively high Re, result in a trade-off where rapid fluid motion outpaces the mixer’s ability to homogenize the contents, as reflected in the drop in MI and HI at higher flow rates.

### 4.5. Robot Length Effect

Figure 7c shows the Mixing Index (MI) and Homogeneity Index (HI) as functions of robot length, tested from 0.75 mm to 2.5 mm in a mixing chamber with a fixed diameter of 3 mm. The distance between the robot and the chamber wall is an essential factor affecting fluid dynamics and mixing performance, with corresponding robot-to-chamber ratios highlighting the relative size of the robot within the chamber.

At the smallest robot length of 0.75 mm, the wall clearance is 1.125 mm on each side, yielding a robot-to-chamber ratio of 0.25. This substantial clearance allows for ample fluid flow around the robot, but the smaller surface area limits the generation of effective mixing forces. In contrast, the maximum robot length of 2.5 mm leaves only 0.25 mm of clearance on each side, with a robot-to-chamber ratio of 0.83. This minimal clearance restricts fluid flow and limits mixing efficiency, even though the larger robot surface could theoretically enhance mixing through increased interaction area. An intermediate length of 1.25 mm, which provides a 0.875 mm clearance on each side and a robot-to-chamber ratio of 0.42, demonstrates the highest mixing performance, achieving MI and HI values of 99.486% and 96.943%, respectively. This balance in robot size and clearance optimizes fluid flow around the robot while maintaining adequate surface area to enhance mixing forces.

## 5. Conclusions

This study demonstrates the effectiveness of magnetically levitated, untethered propeller robots in enhancing fluid mixing within microfluidic systems. By achieving rotational speeds up to 8602.4 rpm, the proposed system allows for rapid and efficient mixing over short distances, presenting a significant advantage over traditional microfluidic mixers. This enhanced control over mixing dynamics is made possible by independently adjusting the propeller’s orientation and velocity through magnetic interactions, making the approach particularly advantageous for lab-on-a-chip applications. Such systems often require precise and efficient mixing for applications like biological sample analysis, where the ability to achieve homogeneity quickly is crucial for reliable results.

A key strength of this system lies in its ability to introduce strong longitudinal forces and torques on the propeller robot, facilitated by its orientation capabilities. These forces enable rapid sample homogenization at the nano-level, addressing a common limitation in passive micromixing strategies. The optimal orientation of the propeller in the roll direction has been shown to increase mixing performance, reaching up to 98.94% efficiency (and up to 99.36% at 6000 rpm) with a low diffusion coefficient and a relatively small output port, demonstrating the method’s potential effectiveness in practical applications.

However, there are limitations to this study that future work should address. Firstly, the model operates under isothermal conditions, excluding potential temperature effects. In microfluidic systems, temperature gradients can significantly affect fluid properties such as viscosity and diffusivity, which, in turn, influence mixing efficiency. Future studies should consider incorporating temperature as a variable to simulate conditions more realistically aligned with experimental setups. Secondly, the assumption of Newtonian fluid behavior, while convenient, may limit the applicability of the model to non-Newtonian fluids, such as many biological samples, which exhibit complex flow characteristics that can alter mixing dynamics. Expanding the model to account for non-Newtonian properties would make it more versatile for applications involving a wider variety of fluids.

Furthermore, while this study models magnetic field effects as uniform and idealized, real-world magnetic fields are often non-uniform, especially when interacting with other magnetic materials. This non-uniformity can impact the stability and control of the microrobots’ motion. Future research could improve the model by including more realistic magnetic field distributions and considering potential distortions, thereby increasing the robustness and reliability of the system in experimental environments. Additionally, while we explored a range of Reynolds numbers, propeller speeds, and levitation heights, the study’s conditions were limited to a specific microchannel geometry and fluid flow configuration. Investigating different channel geometries, propeller shapes, and multiple interacting robots would provide further insights into scalability and adaptability in complex microfluidic networks.

As a result of this work, magnetically levitated microrobots can be used as efficient mixers in microfluidic systems, achieving high efficiencies with rapid mixing rates. Future research should focus on refining the operational dynamics through variations in influential parameters, such as channel size, propeller geometry, and a wider range of Reynolds numbers, to enable simulations across different flow regimes, including creeping, laminar, transition, and turbulent flows. Such advancements would broaden the applicability of this micromixing approach, potentially transforming the design and functionality of microfluidic mixers for diverse lab-on-a-chip and biomedical applications. Furthermore, integrating adaptive sensing mechanisms to dynamically control microrobot movement could enhance the system’s real-time adaptability and mixing efficiency in a wide array of microfluidic scenarios.

## Figures and Tables

**Table 1:** Parameter settings used in COMSOL simulations.

Parameter	Value(s)	Unit
Inlet concentration	$c_{1}$ = 0, $c_{2}$ = 1	mol/m^3^
Wall effects	No slip; $u_{wall}$ = 0	mm/s
Outlet pressure	$P_{o}$ = 0	Pa
Species flow rate ratio	$R_{AB}$ = 1	-
Remanent flux density	*B* = 1.4	T
Reynolds numbers (*Re*)	0.01, 0.1, 1, 10, 100, 1000	-
Propeller speeds	0, 60, 120, 180, 300, 600, 900, 1200, 1500, 3000, 6000	rpm
Robot lengths	0.75, 1, 1.25, 1.5, 1.75, 2, 2.25, 2.5	mm

## Data Availability

Supporting information is available from the corresponding authors upon request.

## Supplementary Materials

The following supporting information can be downloaded at: https://www.mdpi.com/article/10.3390/mi16010052/s1, Table S1. Mixing Efficiency, Standard Deviation Percentage (STD%), Homogeneity Index (HI), and Quality Index across Different Levitation Heights and Panels over Time. Table S2. Mixing Efficiency, Standard Deviation Percentage (STD%), Homogeneity Index (HI), and Quality Index across Different θ and Panels over Time. Table S3. Mixing Efficiency, Standard Deviation Percentage (STD%), Homogeneity Index (HI), and Quality Index across Different ϕ and Panels over Time.

## Abbreviations

The following abbreviations are used in this manuscript:

MDPI	Multidisciplinary Digital Publishing Institute
DOAJ	Directory of open access journals
TLA	Three letter acronym
LD	Linear dichroism
