# Supplementary material for: Enhanced Fluid Mixing in Microchannels Using Levitated Magnetic Microrobots: A Numerical Study
Source: Micromachines (Basel). 2024 Dec 31;16(1):52. doi: 10.3390/mi16010052 (PMC11767584; doi:10.3390/mi16010052)
Supplement: Supplementary file 1 [file micromachines-16-00052-s001.zip › micromachines-3357091-supplementary.pdf]

Enhanced Fluid Mixing in Microchannels Using Levitated Magnetic Microrobots: A Numerical Study

Ali Anil Demircali, Abdurrahim Yilmaz*, Huseyin Uvet*

Table S1 : Mixing Efficiency, Standard Deviation Percentage (STD%), Homogeneity Index (HI), and Quality Index across Different Levitation Heights and Panels over Time

		PANEL I						PANEL II						PANEL III					
	**t(s)**	**25**	**50**	**100**	**150**	**200**	**250**	**25**	**50**	**100**	**150**	**200**	**250**	**25**	**50**	**100**	**150**	**200**	**250**
Mixing Eff	**0.00**	99.369	99.437	99.110	99.328	98.250	99.890	99.801	99.660	99.603	99.911	99.501	98.943	99.821	99.210	99.688	99.703	99.890	99.200
	**0.25**	99.629	99.978	99.548	96.411	96.851	99.293	96.122	95.466	99.817	98.883	99.708	97.900	93.092	92.690	97.581	95.983	96.689	95.571
	**0.50**	99.538	99.402	99.825	96.589	97.438	99.140	99.636	99.665	99.686	97.259	97.661	98.992	97.073	97.745	99.629	99.232	99.711	97.664
	**1.00**	99.458	99.662	99.954	97.898	98.584	98.156	99.936	99.689	99.463	98.268	98.784	98.062	99.747	99.607	99.447	98.163	98.575	98.243
	**1.50**	99.556	99.837	99.806	98.728	99.521	97.581	99.858	99.454	99.293	99.261	99.722	97.411	99.781	99.405	99.269	99.169	99.545	97.538
	**2.00**	99.610	99.968	99.715	99.427	99.955	97.152	99.795	99.297	99.207	99.914	99.680	96.999	99.712	99.237	99.151	99.891	99.762	97.064
	**2.50**	99.661	99.935	99.648	99.716	99.544	96.937	99.751	99.203	99.130	99.666	99.295	96.734	99.663	99.121	99.076	99.645	99.322	96.761
	**3.00**	99.679	99.828	99.648	99.940	99.363	96.741	99.728	99.142	99.130	99.401	99.056	96.565	99.631	99.059	99.076	99.349	99.044	96.566
STD %	**0.00**	48.376	50.378	47.990	49.403	47.664	41.683	51.835	52.898	53.057	50.712	51.737	44.702	46.963	49.217	48.318	49.832	47.085	40.132
	**0.25**	2.205	2.242	2.174	2.450	2.924	1.415	4.365	3.753	1.090	3.749	4.546	3.502	19.006	18.735	3.333	17.357	17.160	14.035
	**0.50**	2.176	2.069	2.134	2.294	2.559	1.310	1.023	0.921	1.153	1.249	1.638	0.614	3.049	3.289	0.966	3.232	3.026	2.193
	**1.00**	2.230	2.037	2.113	2.208	2.425	1.298	1.182	1.021	1.142	1.311	1.656	0.638	0.947	0.822	0.995	1.152	1.515	0.522
	**1.50**	2.187	2.030	2.110	2.163	2.388	1.245	1.186	1.008	1.136	1.275	1.589	0.615	1.023	0.852	0.983	1.124	1.477	0.512
	**2.00**	2.224	2.014	2.100	2.116	2.312	1.254	1.185	1.003	1.128	1.228	1.551	0.595	1.023	0.837	0.977	1.080	1.430	0.493
	**2.50**	2.184	2.013	2.102	2.106	2.314	1.219	1.179	0.993	1.127	1.211	1.520	0.587	1.019	0.831	0.970	1.049	1.403	0.483
	**3.00**	2.219	2.007	2.102	2.073	2.262	1.236	1.180	0.995	1.127	1.188	1.508	0.579	1.017	0.824	0.970	1.032	1.383	0.477
Homogenity	**0.00**	51.930	49.900	52.430	50.260	51.490	58.270	48.060	47.280	46.730	49.330	48.000	54.820	53.120	51.170	51.530	50.320	52.860	59.540
	**0.25**	97.800	97.760	97.820	97.460	96.980	98.600	95.800	96.410	98.910	96.290	95.440	96.570	82.220	82.540	96.750	83.310	83.390	86.560
	**0.50**	97.810	97.920	97.860	97.620	97.370	98.700	98.980	99.080	98.850	98.720	98.320	99.390	97.040	96.780	99.040	96.740	96.970	97.860
	**1.00**	97.760	97.960	97.890	97.740	97.540	98.730	98.820	98.980	98.860	98.670	98.320	99.370	99.060	99.180	99.010	98.830	98.460	99.490
	**1.50**	97.800	97.970	97.890	97.810	97.600	98.780	98.820	99.000	98.870	98.720	98.410	99.400	98.980	99.150	99.020	98.870	98.520	99.500
	**2.00**	97.770	97.990	97.910	97.870	97.690	98.780	98.820	99.000	98.880	98.770	98.450	99.420	98.980	99.170	99.030	98.920	98.570	99.520
	**2.50**	97.810	97.990	97.910	97.890	97.700	98.820	98.820	99.010	98.880	98.790	98.490	99.430	98.980	99.180	99.040	98.950	98.610	99.530
	**3.00**	97.770	98.000	97.910	97.930	97.750	98.800	98.820	99.010	98.880	98.820	98.510	99.440	98.990	99.180	99.040	98.970	98.630	99.540
Quality index	**0.00**	75.649	74.669	75.770	74.794	74.870	79.080	73.930	73.470	73.167	74.620	73.751	76.882	76.470	75.190	75.609	75.011	76.375	79.370
	**0.25**	98.714	98.869	98.684	96.936	96.916	98.947	95.961	95.938	99.364	97.586	97.574	97.235	87.656	87.615	97.165	89.647	90.040	91.065
	**0.50**	98.674	98.661	98.842	97.105	97.404	98.920	99.308	99.372	99.268	97.990	97.990	99.191	97.056	97.262	99.335	97.986	98.341	97.762
	**1.00**	98.609	98.811	98.922	97.819	98.062	98.443	99.378	99.335	99.161	98.469	98.552	98.716	99.404	99.393	99.229	98.497	98.518	98.867
	**1.50**	98.678	98.903	98.848	98.269	98.561	98.181	99.339	99.227	99.082	98.990	99.066	98.406	99.380	99.278	99.144	99.019	99.033	98.519
	**2.00**	98.690	98.979	98.812	98.649	98.823	97.966	99.308	99.149	99.043	99.342	99.065	98.209	99.346	99.203	99.090	99.406	99.166	98.292
	**2.50**	98.736	98.962	98.779	98.803	98.622	97.878	99.286	99.106	99.005	99.228	98.893	98.082	99.322	99.151	99.058	99.297	98.966	98.145
	**3.00**	98.724	98.914	98.779	98.935	98.556	97.770	99.274	99.076	99.005	99.110	98.783	98.002	99.310	99.119	99.058	99.160	98.837	98.053

Table S2 : Mixing Efficiency, Standard Deviation Percentage (STD%), Homogeneity Index (HI), and Quality Index across Different θ and Panels over Time

		PANEL I					PANEL II					PANEL III				
	**t(s)**	**0°**	**1°**	**2°**	**3°**	**4°**	**0°**	**1°**	**2°**	**3°**	**4°**	**0°**	**1°**	**2°**	**3°**	**4°**
Mixing Eff	**0.00**	99.328	90.992	91.210	91.207	90.983	99.911	99.081	99.097	99.097	99.090	99.703	98.814	98.640	98.640	98.753
	**0.25**	96.411	89.698	89.140	89.087	88.869	98.883	94.317	94.449	94.306	94.528	95.983	98.494	98.603	98.507	98.688
	**0.50**	96.589	91.069	91.231	91.291	91.184	97.259	91.565	91.472	91.496	91.533	99.232	93.667	93.671	93.662	93.726
	**1.00**	97.898	94.070	94.332	94.338	94.272	98.268	94.322	94.305	94.330	94.380	98.163	94.047	94.029	94.063	94.116
	**1.50**	98.728	95.821	96.101	96.138	96.084	99.261	96.322	96.326	96.349	96.420	99.169	96.195	96.210	96.228	96.302
	**2.00**	99.427	96.866	97.120	97.112	97.087	99.914	97.439	97.444	97.448	97.527	99.891	97.455	97.476	97.491	97.573
	**2.50**	99.716	97.350	97.596	97.618	97.580	99.666	98.040	98.007	98.016	98.114	99.645	98.169	98.168	98.171	98.272
	**3.00**	99.940	97.693	97.906	97.891	97.887	99.401	98.371	98.335	98.329	98.430	99.349	98.537	98.525	98.527	98.633
STD %	**0.00**	49.403	53.122	53.153	53.153	53.132	51.835	56.707	56.683	56.683	56.703	46.963	54.960	55.401	55.401	55.143
	**0.25**	2.450	4.447	5.456	5.437	5.439	4.365	12.112	12.660	12.662	12.485	19.006	23.910	24.466	24.454	24.335
	**0.50**	2.294	3.187	3.554	3.554	3.479	1.023	2.730	3.031	3.079	2.993	3.049	6.089	6.484	6.451	6.379
	**1.00**	2.208	3.007	3.190	3.205	3.191	1.182	2.765	3.006	3.053	2.979	0.947	2.550	2.765	2.816	2.744
	**1.50**	2.163	2.938	3.133	3.154	3.081	1.186	2.552	2.751	2.800	2.731	1.023	2.413	2.614	2.652	2.587
	**2.00**	2.116	2.823	2.980	3.002	2.979	1.185	2.437	2.654	2.694	2.613	1.023	2.223	2.425	2.468	2.393
	**2.50**	2.106	2.833	3.027	3.044	2.959	1.179	2.362	2.557	2.607	2.535	1.019	2.126	2.329	2.369	2.296
	**3.00**	2.073	2.760	2.922	2.946	2.916	1.180	2.337	2.553	2.593	2.513	1.017	2.064	2.273	2.317	2.236
Homogenity	**0.00**	50.260	41.620	41.720	41.720	41.600	48.060	42.770	42.800	42.800	42.780	53.120	44.380	43.840	43.840	44.160
	**0.25**	97.460	95.040	93.880	93.900	93.880	95.800	87.160	86.600	86.570	86.790	82.220	75.720	75.190	75.180	75.340
	**0.50**	97.620	96.500	96.100	96.110	96.180	98.980	97.020	96.690	96.640	96.730	97.040	93.500	93.080	93.110	93.190
	**1.00**	97.740	96.800	96.620	96.600	96.620	98.820	97.070	96.810	96.760	96.840	99.060	97.290	97.060	97.010	97.080
	**1.50**	97.810	96.930	96.740	96.720	96.790	98.820	97.350	97.140	97.090	97.170	98.980	97.490	97.280	97.240	97.310
	**2.00**	97.870	97.090	96.930	96.910	96.930	98.820	97.500	97.280	97.240	97.320	98.980	97.720	97.510	97.470	97.550
	**2.50**	97.890	97.090	96.900	96.880	96.970	98.820	97.590	97.390	97.340	97.420	98.980	97.830	97.630	97.590	97.660
	**3.00**	97.930	97.180	97.020	96.990	97.020	98.820	97.620	97.400	97.360	97.450	98.990	97.910	97.690	97.650	97.730
Quality index	**0.00**	74.794	66.306	66.465	66.464	66.292	73.985	70.926	70.949	70.949	70.935	76.411	71.597	71.240	71.240	71.457
	**0.25**	96.936	92.369	91.510	91.494	91.375	97.341	90.738	90.525	90.438	90.659	89.102	87.107	86.896	86.844	87.014
	**0.50**	97.105	93.785	93.666	93.701	93.682	98.120	94.292	94.081	94.068	94.132	98.136	93.583	93.376	93.386	93.458
	**1.00**	97.819	95.435	95.476	95.469	95.446	98.544	95.696	95.557	95.545	95.610	98.612	95.668	95.545	95.536	95.598
	**1.50**	98.269	96.375	96.421	96.429	96.437	99.040	96.836	96.733	96.720	96.795	99.074	96.842	96.745	96.734	96.806
	**2.00**	98.649	96.978	97.025	97.011	97.008	99.367	97.470	97.362	97.344	97.423	99.436	97.588	97.493	97.481	97.561
	**2.50**	98.803	97.220	97.248	97.249	97.275	99.243	97.815	97.698	97.678	97.767	99.312	98.000	97.899	97.880	97.966
	**3.00**	98.935	97.436	97.463	97.440	97.453	99.110	97.996	97.867	97.844	97.940	99.170	98.223	98.107	98.089	98.182

Table S3 : Mixing Efficiency, Standard Deviation Percentage (STD%), Homogeneity Index (HI), and Quality Index across Different ϕ and Panels over Time

		PANEL I					PANEL II					PANEL III				
	**t(s)**	**0°**	**1°**	**2°**	**3°**	**4°**	**0°**	**1°**	**2°**	**3°**	**4°**	**0°**	**1°**	**2°**	**3°**	**4°**
Mixing Eff	**0.00**	99.328	99.610	99.661	99.590	99.590	99.911	99.763	99.764	99.709	99.574	99.703	99.645	99.540	99.736	99.854
	**0.25**	96.411	97.545	97.712	96.962	96.962	98.883	94.964	94.816	94.143	93.874	95.983	93.196	92.918	92.542	92.362
	**0.50**	96.589	97.913	98.247	97.461	97.461	97.259	97.507	97.673	97.009	96.851	99.232	96.348	96.371	95.726	95.544
	**1.00**	97.898	97.414	97.846	97.210	97.210	98.268	97.013	97.445	96.829	96.752	98.163	97.086	97.448	96.839	96.732
	**1.50**	98.728	96.858	97.622	96.860	96.860	99.261	96.564	97.172	96.553	96.521	99.169	96.623	97.178	96.571	96.520
	**2.00**	99.427	97.545	97.407	96.741	96.741	99.914	94.964	96.982	96.361	96.358	99.891	96.279	96.971	96.354	96.338
	**2.50**	99.716	97.913	97.335	96.577	96.577	99.666	97.507	96.867	96.251	96.248	99.645	96.071	96.836	96.228	96.218
	**3.00**	99.940	97.414	97.184	96.562	96.562	99.401	97.013	96.768	96.160	96.175	99.349	95.942	96.736	96.137	96.135
STD %	**0.00**	49.403	43.705	43.744	43.702	43.720	51.835	45.688	45.689	45.696	45.706	46.963	41.883	41.889	41.877	41.873
	**0.25**	2.450	1.179	1.377	1.198	1.171	4.365	2.748	2.786	2.831	2.794	19.006	15.644	15.700	15.725	15.555
	**0.50**	2.294	1.134	1.247	1.156	1.156	1.023	0.435	0.422	0.412	0.386	3.049	2.437	2.465	2.504	2.474
	**1.00**	2.208	1.128	1.314	1.188	1.191	1.182	0.421	0.464	0.441	0.430	0.947	0.349	0.350	0.335	0.310
	**1.50**	2.163	1.081	1.216	1.128	1.141	1.186	0.402	0.455	0.433	0.418	1.023	0.319	0.354	0.341	0.321
	**2.00**	2.116	1.179	1.294	1.174	1.176	1.185	2.748	0.449	0.427	0.419	1.023	0.304	0.344	0.332	0.313
	**2.50**	2.106	1.134	1.203	1.121	1.131	1.179	0.435	0.445	0.420	0.410	1.019	0.291	0.338	0.326	0.309
	**3.00**	2.073	1.128	1.283	1.163	1.170	1.180	0.421	0.438	0.424	0.414	1.017	0.295	0.329	0.322	0.305
Homogenity	**0.00**	50.260	56.460	56.400	56.480	56.440	48.060	54.200	54.200	54.170	54.100	53.120	57.970	57.920	58.010	58.070
	**0.25**	97.460	98.850	98.650	98.840	98.870	95.800	97.380	97.350	97.330	97.370	82.220	85.350	85.340	85.370	85.550
	**0.50**	97.620	98.890	98.770	98.870	98.870	98.980	99.580	99.590	99.600	99.630	97.040	97.650	97.620	97.600	97.630
	**1.00**	97.740	98.900	98.710	98.840	98.840	98.820	99.590	99.550	99.570	99.580	99.060	99.660	99.660	99.670	99.700
	**1.50**	97.810	98.950	98.810	98.910	98.890	98.820	99.610	99.560	99.580	99.600	98.980	99.690	99.660	99.670	99.690
	**2.00**	97.870	98.850	98.740	98.860	98.860	98.820	97.380	99.560	99.590	99.600	98.980	99.710	99.670	99.680	99.700
	**2.50**	97.890	98.890	98.830	98.920	98.910	98.820	99.580	99.570	99.600	99.610	98.980	99.720	99.670	99.690	99.700
	**3.00**	97.930	98.900	98.750	98.880	98.870	98.820	99.590	99.580	99.590	99.600	98.990	99.720	99.680	99.690	99.710
Quality index	**0.00**	74.794	78.035	78.031	78.035	78.015	73.985	76.982	76.982	76.940	76.837	76.411	78.808	78.730	78.873	78.962
	**0.25**	96.936	98.198	98.181	97.901	97.916	97.341	96.172	96.083	95.736	95.622	89.102	89.273	89.129	88.956	88.956
	**0.50**	97.105	98.402	98.509	98.166	98.166	98.120	98.543	98.632	98.304	98.240	98.136	96.999	96.996	96.663	96.587
	**1.00**	97.819	98.157	98.278	98.025	98.025	98.544	98.302	98.498	98.199	98.166	98.612	98.373	98.554	98.255	98.216
	**1.50**	98.269	97.904	98.216	97.885	97.875	99.040	98.087	98.366	98.066	98.061	99.074	98.156	98.419	98.121	98.105
	**2.00**	98.649	98.198	98.074	97.801	97.801	99.367	96.172	98.271	97.975	97.979	99.436	97.994	98.320	98.017	98.019
	**2.50**	98.803	98.402	98.083	97.749	97.744	99.243	98.543	98.219	97.926	97.929	99.312	97.895	98.253	97.959	97.959
	**3.00**	98.935	98.157	97.967	97.721	97.716	99.110	98.302	98.174	97.875	97.887	99.170	97.831	98.208	97.914	97.923
